# Predicting Biomass and Yield in a Tomato Phenotyping Experiment Using UAV Imagery and Random Forest

**DOI:** 10.3389/frai.2020.00028

**Published:** 2020-05-08

**Authors:** Kasper Johansen, Mitchell J. L. Morton, Yoann Malbeteau, Bruno Aragon, Samer Al-Mashharawi, Matteo G. Ziliani, Yoseline Angel, Gabriele Fiene, Sónia Negrão, Magdi A. A. Mousa, Mark A. Tester, Matthew F. McCabe

**Affiliations:** ^1^Water Desalination and Reuse Center, King Abdullah University of Science and Technology, Thuwal, Saudi Arabia; ^2^Center for Desert Agriculture, King Abdullah University of Science and Technology, Thuwal, Saudi Arabia; ^3^School of Biology and Environmental Science, University College Dublin, Dublin, Ireland; ^4^Department of Arid Land Agriculture, Faculty of Meteorology, Environment and Arid Land Agriculture, King Abdulaziz University, Jeddah, Saudi Arabia; ^5^Department of Vegetables, Faculty of Agriculture, Assiut University, Assiut, Egypt

**Keywords:** UAV, yield, biomass, tomato plants, salinity, random forest, RGB, multi-spectral

## Abstract

Biomass and yield are key variables for assessing the production and performance of agricultural systems. Modeling and predicting the biomass and yield of individual plants at the farm scale represents a major challenge in precision agriculture, particularly when salinity and other abiotic stresses may play a role. Here, we evaluate a diversity panel of the wild tomato species (*Solanum pimpinellifolium*) through both field and unmanned aerial vehicle (UAV)-based phenotyping of 600 control and 600 salt-treated plants. The study objective was to predict fresh shoot mass, tomato fruit numbers, and yield mass at harvest based on a range of variables derived from the UAV imagery. UAV-based red–green–blue (RGB) imageries collected 1, 2, 4, 6, 7, and 8 weeks before harvest were also used to determine if prediction accuracies varied between control and salt-treated plants. Multispectral UAV-based imagery was also collected 1 and 2 weeks prior to harvest to further explore predictive insights. In order to estimate the end of season biomass and yield, a random forest machine learning approach was implemented using UAV-imagery-derived predictors as input variables. Shape features derived from the UAV, such as plant area, border length, width, and length, were found to have the highest importance in the predictions, followed by vegetation indices and the entropy texture measure. The multispectral UAV imagery collected 2 weeks prior to harvest produced the highest explained variances for fresh shoot mass (87.95%), fruit numbers (63.88%), and yield mass per plant (66.51%). The RGB UAV imagery produced very similar results to those of the multispectral UAV dataset, with the explained variance reducing as a function of increasing time to harvest. The results showed that predicting the yield of salt-stressed plants produced higher accuracies when the models excluded control plants, whereas predicting the yield of control plants was not affected by the inclusion of salt-stressed plants within the models. This research demonstrates that it is possible to predict the average biomass and yield up to 8 weeks prior to harvest within 4.23% of field-based measurements and up to 4 weeks prior to harvest at the individual plant level. Results from this work may be useful in providing guidance for yield forecasting of healthy and salt-stressed tomato plants, which in turn may inform growing practices, logistical planning, and sales operations.

## Introduction

Along with growing populations and the challenges of climate change, salt-stress presents as a major threat to global food production. While soil salinity in irrigated agriculture is a global concern, it is particularly so in arid and semiarid climates (Pitman and Lauchli, 2002; Rao et al., 2013; Machado and Serralheiro, 2017). Breeding of crop cultivars with improved salt tolerance represents one potential pathway toward improving food and water security (Hickey et al., 2019; Johansen et al., 2019a). To do this requires the identification of salt-tolerant genotypes/accessions, whose tolerance traits can then be introgressed into commercial varieties (Munns and Tester, 2008; Messerer et al., 2018; Morton et al., 2019). In order to identify the potential salt tolerance of plant accessions, phenotyping and related approaches that can effectively map, monitor, and predict plant biophysical and biochemical properties are required (Johansen et al., 2019a). Two indicators of plant response that can offer insight into performance are biomass and yield. While the effects of salinity are to generally reduce a plant's biomass and yield, what is not well-understood is how salt stress affects the ability to predict these variables ahead of harvest time (Flowers and Flower, 2005; Verslues et al., 2006; Stavridou et al., 2017; Johansen et al., 2019a,b).

Measurements of biomass provide information on a plant's ability to capture sunlight, water, and minerals, and the rates at which it can turn these into physical growth (Johansen et al., 2019b). It is also useful in informing the amount and timing of fertilizer, pesticides, and water to be applied to optimize crop performance and improve agricultural management practices (Jaleel et al., 2009; Bendig et al., 2015): key metrics behind the concept of precision agriculture. Predicting yield prior to harvest can facilitate logistical planning and scheduling of field and harvest operations, e.g., fruit picking, storage, packaging, and transportation (Robson et al., 2017), as well as help in financial planning and management. In recent years, predicting biomass and yield (along with other biophysical and biochemical properties) through various types of sensing technologies has become a focus for both precision agriculture and smart farming. While precision agriculture attempts to observe, measure, and respond to inter- and intrafield crop variability, smart farming encompasses a focus on agricultural systems management via big data analytics, using context and situation awareness, often in real time (Zhang et al., 2002; Gebbers and Adamchuk, 2010; Wolfert et al., 2017).

Both precision agriculture and smart farming require large amounts of data to ensure informed decision-making at the specific plant, tree, or plot level. Data required to drive timely or near real-time information-based decisions may be obtained using a range of remote sensing platforms, as well as in-field robotics and sensing technologies (Wolfert et al., 2017). However, satellite-based remote sensing is often unable to provide the spatial resolution required for per-plant or per-tree assessment, with timely data acquisition often affected by cloud cover or other adverse atmospheric conditions (Nevavuori et al., 2019). Airborne remote sensing remains costly, especially if used for high-frequency assessment of growth patterns and other crop parameters relying on multitemporal data (Koh and Wich, 2012). Field-based robotics have proved useful for fruit counting, fruit ripening assessment, flower identification, yield prediction, and measurements of three-dimensional structure (Underwood et al., 2016; Bargoti and Underwood, 2017; Wang Z. et al., 2018; Wendel et al., 2018; Westling et al., 2018) but are generally restricted to smaller areas with no ground obstacles to hinder access (Kragh and Underwood, 2019). At the other end of the spectrum, it can be very time consuming, labor intensive, and subjective to consistently collect field data suitable for predicting biomass and yield at harvest (Sugiura et al., 2015; Holman et al., 2016). The divide between space- and ground-based data collection has recently been filled by the use of unmanned aerial vehicles (UAVs), which provide a means for efficient, regular, and flexible collection of imagery at very high spatial resolutions, suitable for regular assessment of crops, their properties, and stress factors (Gil-Docampo et al., 2018). Deployment of UAVs for data collection also reduces the requirement for human-based on-site observations (and potential for investigator bias), increases safety and access, and facilitates the implementation of management practices in the agricultural sector (Shi et al., 2016; Barbedo, 2019).

Despite the commercial importance of tomatoes, relatively few studies have assessed the potential for using UAV-based imagery for modeling their biomass and yield [annual global production is ~171 million tons; (FAOSTAT, 2017)]. Senthilnath et al. (2016) used two UAV-derived images to delineate and classify tomato fruits on individual plants but found that many fruits were omitted, as they were visually occluded by leaves and stalks. Johansen et al. (2019a) used a time series of RGB and multispectral UAV imagery to accurately monitor phenotypic traits of individual tomato plants, including plant area, plant projective cover, condition, and growth rate, and used these variables to successfully identify tomato plant accessions that performed the best in terms of yield. Moeckel et al. (2018) estimated crop height and biomass of eggplant, tomato, and cabbage plants from a time series of five red, green, and blue (RGB) UAV-based data sets and found measured crop height to correlate well with biomass when using random forest and support vector regression. Johansen et al. (2019b) provided some initial findings for using UAV imagery to predict biomass and yield at harvest. However, this initial work only assessed prediction accuracies of individual plants from RGB imagery and excluded an evaluation on variable importance for the prediction models. This paper significantly expands on the interpretation of these preliminary results and further explores the use of multispectral imagery, variable importance, model predictions of biomass, fruit numbers, and yield mass, and a comparison of model results for control and salt-treated plants.

The use of UAVs for plant phenotyping purposes has witnessed their application for plant height assessment (Hu et al., 2018; Wang X. et al., 2018), genotype performance under low nitrogen conditions (Buchaillot et al., 2019), crop growth monitoring (Holman et al., 2016), among many others (Yang et al., 2017). While UAV imagery has only been exploited to a limited extent for predicting biomass and yield of tomato plants, it has been applied to assess biomass and yield of other crops. For instance, Fathipoor et al. (2019) used RGB UAV imagery acquired at the mid-season growth stage to predict corn forage yield using a combination of plant height and vegetation indices for partial least square regression. Ballesteros et al. (2018) used height derived from UAV-based RGB imagery as well as green canopy cover and canopy volume to estimate the biomass of onion, with canopy volume found to be particularly informative. Han et al. (2019) used various predictor variables, including plant height, canopy shape, and vegetation indices, to predict aboveground biomass of maize from UAV-based imagery and achieved the best results using a random forest model. Nevavuori et al. (2019) used convolutional neural networks (CNN) to build a model for predicting yields of wheat and barley fields using multispectral UAV imagery. They found that yield prediction errors were lower for UAV data acquired early in the growth season than using data acquired later and closer to harvest. Other examples of UAV-based studies using machine learning approaches for biomass estimation include the mapping of wheat (Lu et al., 2019), grass sward (Nasi et al., 2018), rice (Jiang et al., 2019), and maize (Han et al., 2019). Related crop yield estimation studies include the mapping of oilseed rape (Peng et al., 2019), barley (Escalante et al., 2019), rice (Yang et al., 2019), and cotton (Zou et al., 2018). Apart from the work by Moeckel et al. (2018) and Johansen et al. (2019a,b), no other research was identified using UAV-based time series for the prediction of biomass and yield of tomato plants at harvest.

The UAV-based studies on yield and biomass mapping reviewed above used a variety of artificial intelligence approaches, including both machine learning and deep learning techniques. In fact, Liakos et al. (2018) identified yield prediction as one of the most common applications of machine learning in agriculture. Although big data analysis is becoming more common in the agricultural sector (Kamilaris et al., 2017), obtaining the required data volumes of suitable quality needed for large-scale application of machine learning approaches remains a challenge. Still, more traditional and established machine learning approaches are often beneficial for smaller scale studies, where interpretability may also be important. From the selection of machine learning algorithms used in agricultural UAV-based studies, the random forest approach has been regularly identified as producing the best results, which is commonly attributed to its lower sensitivity to data skewness and prevention of model overfitting (e.g., Moeckel et al., 2018; Han et al., 2019; Lu et al., 2019).

Overall, the objectives of this research were three-fold: (1) to predict biomass (fresh shoot mass) and yield (tomato fruit numbers and yield mass) at harvest from a time series of RGB UAV-based imagery; (2) to determine if prediction accuracies varied between control and salt-treated plants; and (3) to compare results of RGB and multispectral UAV imagery collected 1 and 2 weeks before harvest. To do this, a random forest machine learning approach was employed to predict biomass and yield for both control and salt-treated plants using a time series of six RGB and two multispectral UAV image data sets collected prior to harvest. An ability to forecast at-harvest biomass and yield would offer growers a capacity to identify and understand biomass and yield variability in areas affected by salinity and could be used to optimize per plant inputs during the growing season, while also informing logistical and sales related operations.

## Materials and Methods

### Study Area and Experimental Design

**Figure 1:**
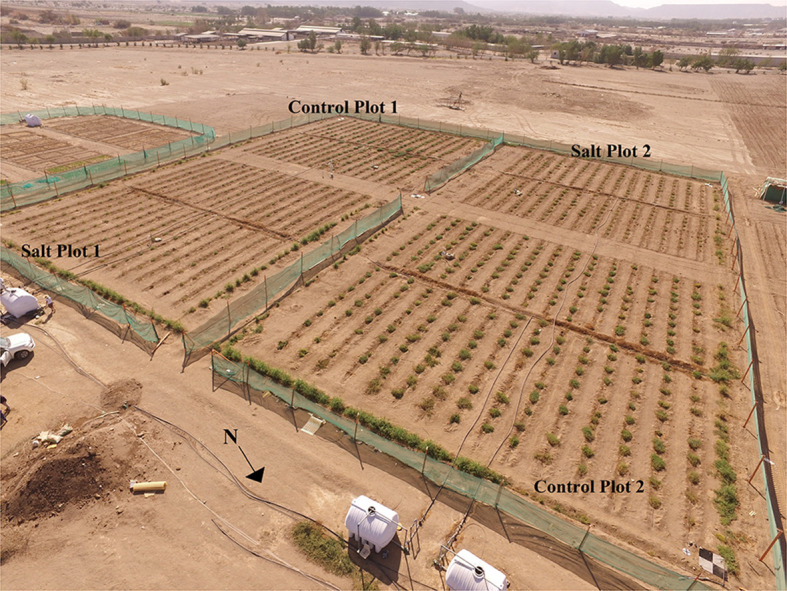
Setup of the tomato plant trial with 300 plants in each of the two control and two salt-treated plots. The plant trial covered an area of ~75 × 75 m, with each plot of 300 plants being 30 × 30 m.

The study area was located at the King Abdulaziz University Agricultural Research Station in Hada Al-Sham (21.7967°N, 39.7264°E), about 60 km east of Jeddah in the Makkah region of Saudi Arabia. The area receives an annual rainfall of <100 mm and has a predominantly sandy loam soil type. A tomato field-trial experiment was initiated in November 2017, with the field arrangement consisting of four 30 × 30 m plots. In each of the four plots, 15 rows of 20 tomato plants were planted, producing a combined total of 1,200 plants (Figure 1). These 1,200 plants included 200 different genotypes, consisting of 199 *Solanum pimpinellifolium* accessions and one *Solanum lycopersicum* accession (the commercial tomato, Heinz 1706). The 199 *S. pimpinellifolium* accessions originated from different parts of Peru and Ecuador.

Seeds for these accessions were propagated at King Abdullah University of Science and Technology (KAUST) (Johansen et al., 2019a). Using these, the tomato plants were sown at a greenhouse nursery at KAUST a month before transplanting, which occurred on November 1-2, 2017. Plants were allocated into two control and two salt-treated plots, following a randomized design (Figure 1). Three replicates of the 200 accessions were planted in each of the two treatments. The two control plots were irrigated with low salinity water (27 mM NaCl, 900-1,000 ppm) throughout the growing season. The two salt-treated plots were irrigated with saline water of 127 mM NaCl (4,500 ppm) from November 14, 2017, 197 mM NaCl (7,000 ppm) from December 4, 225 mM NaCl (8,000 ppm) from December 10, 254 mM NaCl (9,000 ppm) from December 18, and 183 mM NaCl (6,500 ppm) from January 12, 2018 until harvest, which occurred between January 16-22 (Johansen et al., 2019a). Drip irrigation was applied once in the morning for 10 min and again in the evening for 10 min until November 9, then for 15 min in the morning and evening until December 17, and 30 min in the morning and evening until harvest, in response to the increasing irrigation requirements of growing plants. Weeds within each plot were manually removed before each of the UAV flights. During the growing season, maximum day and minimum night temperatures ranged from 27 to 37°C and 12 to 24°C, respectively. During the growing season, no rainfall was recorded, but several sandstorms occurred, with the most severe and damaging on December 8 and 16, 2017. After each sandstorm, farm employees washed the plants with non-saline water to remove dust from the leaves (Johansen et al., 2019a).

### Field Data Collection

Field and UAV data were collected on November 23 and 30, December 6 and 20, 2017, and January 7 and 14, 2018, i.e., 1, 2, 4, 6, 7, and 8 weeks before harvest. Five GCPs were deployed for geo-referencing the UAV imagery and their coordinates measured at the planting date (November 2) using a Leica GS10 base station with an AS10 antenna and a Leica GD15 smart antenna as a rover (Leica Geosystems, St. Gallen, Switzerland). GCPs were placed at the center and at each of the four corners of the study site. All collected Global Navigation Satellite System data were postprocessed using the Leica Geo Office software (Leica Geosystems, St. Gallen, Switzerland). Six near-Lambertian panels in white, four shades of gray, and in black (Johansen et al., 2018, 2019a) were placed within the field and measured with an ASD FieldSpec4 spectrometer (Malvern Panalytical, Malvern, UK) for radiometric calibration of the collected imagery.

Between January 16 and 26, fresh shoot mass, fruit numbers, and yield mass were measured for all tomato plants remaining at harvest. The fresh shoot mass, including aboveground plant material and fruit, was measured first. Fresh shoot mass ranged from 17 to 5,402 g per plant, averaging 715 g/plant (Table 1). Fruit numbers were manually counted for both mature (fruits with some redness) and immature (green fruits) fruits >3 mm in diameter on each plant. The fruit was subsequently weighed for each plant. For plants with >1 kg shoot mass, a representative sample of the whole plant was selected to count and weigh all fruits >3 mm in diameter. To estimate the fruit numbers and their weight for the whole plant, the sample was used for extrapolation and multiplied by the weight ratio of the whole plant and the selected sample. Based on all harvested plants, the number of tomatoes ranged from 1 to 3,349 per plant, averaging 532 fruits/plant. Yield mass ranged from 0.1 to 1,433 g/plant, averaging 227 g/plant (Johansen et al., 2019a). The total number of observations at harvest for fresh shoot mass, fruit number, and yield mass were 1,027 (514 control and 513 salt treated), 980 (496 control and 484 salt treated), and 979 (497 control and 482 salt treated) plants, respectively (Table 1). The present study only examines those plants that survived until harvest.

**Table 1:** Minimum, maximum, and mean values and the total number of observations (obs.) as well as control and salt observations of fresh shoot mass, fruit numbers, and yield mass.

	**Minimum**	**Maximum**	**Mean**	**Total obs**.	**Control obs**.	**Salt obs**.
Fresh shoot mass	17 g/plant	5,402 g/plant	715 g/plant	1,027	514	513
Fruit numbers	1 fruit/plant	3,349 fruits/plant	532 fruits/plant	980	496	484
Yield mass	0.1 g/plant	1,433 g/plant	272 g/plant	979	497	422

*Note that only fruits >3 mm in diameter were included*.

### UAV Data Collection and Processing

A gimbal-stabilized Zenmuse X3 camera (Dà-Jiāng Innovations, Shenzhen, China) installed on a DJI Matrice 100 (Dà-Jiāng Innovations, Shenzhen, China) quadcopter was used to collect RGB imagery for all of the six field campaigns, i.e., 1, 2, 4, 6, 7, and 8 weeks before harvest. The Zenmuse X3 camera has a Sony EXMOR 1/2.3″ complementary metal-oxide semiconductor (CMOS) sensor with the full width at half-maximum being ~400–510 nm for the blue band, 480–600 nm for the green band, and 580-700 nm for the red band (Sato et al., 2016). The Parrot Sequoia sensor (Parrot SA, Paris, France), also installed on the DJI Matrice 100, was used to collect coincident multispectral green (530–570 nm), red (640–680 nm), red edge (730–740 nm), and near-infrared (NIR) (770–810 nm) imagery for the last two campaigns, i.e., 1 and 2 weeks before harvest. All UAV campaigns occurred under cloud-free conditions at around solar noon. The Universal Ground Control Station (UgCS) Client application (SPH Engineering, SIA, Riga, Latvia) was used for flight planning. For each UAV campaign, the image data were collected at an altitude of 13 m above ground level and at a speed of 2 m/s. This produced a forward overlap and sidelap of 78 and 82%, respectively, for the RGB imagery (3-s image intervals) and 83 and 68%, respectively, for the multispectral imagery (1-s image intervals) (Johansen et al., 2019a). A geo-referenced orthomosaic and digital surface model (DSM) were produced at a pixel size of 0.5 and 1.12 cm for the RGB and multispectral imagery, respectively, using Agisoft PhotoScan (Agisoft LLC, St. Petersburg, Russia). A digital terrain model (DTM), produced from RGB UAV imagery collected of the study site before planting, was subtracted from the DSMs to produce canopy height models (CHMs) for each of the six UAV campaign (Johansen et al., 2019a).

Buchaillot et al. (2019), Madec et al. (2017), and Singh et al. (2019) used RGB UAV imagery for phenotyping maize and wheat and argued that no radiometric correction of the UAV imagery was required, at least when using point cloud information for plant height measurements and field-based RGB imagery acquired coincidently with the UAV data. However, it is considered good practice to radiometrically calibrate UAV imagery to remove or normalize effects of sun-object-sensor geometry and illumination conditions (Tmusic et al., 2020). The empirical line calibration method has become a standard approach for UAV-based studies to correct the imagery to at-surface reflectance using either natural features (Lelong et al., 2008) or other types of commercial (e.g., spectralon) or more cost-effective Lambertian panels, e.g., made from masonite, plywood, or ethylene-vinyl acetate (Wang and Myint, 2015; Jeong et al., 2018; Barreto et al., 2019; Tmusic et al., 2020). An empirical line correction, produced between field-derived spectrometer measurements and the digital numbers of the radiometric calibration panels within the multispectral and RGB orthomosaics, was used to convert the orthomosaics to at-surface reflectance (Ahmed et al., 2017; Johansen et al., 2018). For the empirical line corrections, the coefficients of determination (*R*^2^) were >0.98 for all band combinations and data sets. While the multispectral imagery produced a linear empirical line, an exponential relationship was required for the RGB imagery. An exponential relationship of the empirical line correction for RGB UAV imagery was also identified by Jeong et al. (2018).

### Delineation of Tomato Plants

The eCognition Developer 9.3 software (Trimble, Munich, Germany) was used to develop an object-based rule set to delineate all individual tomato plants within the six RGB and two multispectral orthomosaics. After a fine-scale multiresolution segmentation, vegetation indices, and spectral band combinations were used to classify objects, representing the green parts of the tomato plants. A region-growing algorithm was subsequently applied to expand tomato plant objects into neighboring unclassified objects using more relaxed vegetation index thresholds. Unclassified objects enclosed by tomato plant objects were merged with the enclosing tomato plant objects. To remove small incorrectly classified tomato plant objects, an area threshold of <150 cm^2^ was applied. Remaining tomato plants were then resized using a number of growing and shrinking processes and the use of the produced CHM to adjust the edges of the tomato plant objects. The delineation results of the six RGB and two multispectral orthomosaics were visually assessed and manually edited if necessary. A more detailed description of the object-based approach can be found in Johansen et al. (2019a).

### Extraction of Image Variables

Based on the delineated plants, shape, spectral, and texture variables were extracted from both the RGB and multispectral UAV imagery. For the RGB imagery, the extracted variables included the three RGB bands, the Green–Red Index (Motohka et al., 2010), nine shape features (border length, width, length, length/width ratio, elliptic fit, shape index, compactness, roundness, and border index) exported directly from the eCognition Developer software, four gray-level co-occurrence textural measures [homogeneity, contrast, entropy, and dissimilarity; (Haralick et al., 1973)] based on the three spectral bands and the Green–Red Index, maximum plant height, and the standard deviation of maximum height (see Table 2). For the multispectral UAV imagery, the extracted variables included the four spectral bands (green, red, red edge, and NIR), six vegetation indices (Robson et al., 2017) (Table 3), nine shape features, four gray-level co-occurrence textural measures [homogeneity, contrast, entropy, and dissimilarity; (Haralick et al., 1973; Lofstedt et al., 2019)] based on the four spectral bands and the normalized difference vegetation index (NDVI), as well as maximum height and the standard deviation of maximum height (see Table 2).

**Table 2:** Variables extracted (gray fields) from the red–green–blue (RGB) (35 in total) and multispectral (46 in total) unmanned aerial vehicle (UAV) imagery for each individual tomato plant.

**Image-derived variables**	**MS**	**RGB**	**Image-derived variables**	**MS**	**RGB**
Border length			Homogeneity blue		
Width			Homogeneity green		
Length			Homogeneity Green–Red Index		
Length/width ratio			Homogeneity red		
Elliptic fit			Homogeneity red edge		
Shape index			Homogeneity NIR		
Compactness			Homogeneity NDVI		
Roundness			Contrast blue		
Border index			Contrast green		
Area			Contrast Green–Red Index		
Maximum height			Contrast red		
Standard deviation height			Contrast red edge		
Blue			Contrast NIR		
Green			Contrast NDVI		
Red					
Red edge			Entropy green		
NIR			Entropy Green–Red Index		
Green–Red Index			Entropy red		
NDVI			Entropy red edge		
RENDVI			Entropy NIR		
NDRE			Entropy NDVI		
NIRRENDVI			Dissimilarity blue		
Green NDVI			Dissimilarity green		
Standard deviation blue			Dissimilarity Green–Red Index		
Standard deviation green			Dissimilarity red		
Standard deviation red			Dissimilarity red edge		
Standard deviation red edge			Dissimilarity NIR		
Standard deviation NIR			Dissimilarity NDVI		

**Table 3:** Vegetation indices calculated from the multispectral unmanned aerial vehicle (UAV) imagery and extracted for each individual tomato plant.

**Vegetation Indices**	**Equation**	**References**
Green–Red index	(Green – red)/(Green + red)	Motohka et al., 2010
NDVI	(NIR – red)/(NIR + red)	Rouse et al., 1973
RENDVI	(Red edge – red)/(red edge + red)	Sims and Gamon, 2002b
NDRE	(NIR – red edge)/(NIR + red edge)	Jorge et al., 2019
NIRRENDVI	{[(NIR + red edge)/2] – red}/{[(NIR + red edge)/2] + red}	Xie et al., 2018
Green NDVI	(NIR – green)/(NIR + green)	Gitelson and Merzlyak, 1998

Image-based texture measures have been found useful for UAV-based biomass estimation in other studies (Zheng et al., 2019) and may provide additional spatial information useful for improving classification results (Johansen et al., 2007). To achieve directional invariance, the sum of all four directions (0, 45, 90, and 135°) were calculated before the texture calculation. The calculation of texture [following (Gil-Docampo et al., 2018)] was independent of the image data bit depth, as the dynamic range was interpolated to 8 bit before evaluating the co-occurrence (Trimble eCognition Developer, 2017; Lofstedt et al., 2019). All extracted per-plant image variables from all eight image data sets (six RGB and two multispectral) were then used to assess their linear, exponential, logarithmic, and second-order polynomial correlation with the corresponding field-derived measurements of biomass, fruit numbers, and yield mass. Those variables with an *R*^2^ < 0.10 in all four correlations were omitted from further analysis, as they were considered insignificant. The remaining variables were used for predicting biomass and yield using a random forest machine learning approach.

### Random Forest Modeling and Analysis

The random forest machine learning approach has been applied widely in ecological studies (e.g., Prasad et al., 2006; Cutler et al., 2007) and in various remote-sensing-based analyses (e.g., Belgiu and Dragut, 2016; Shi and Yang, 2016; Ma et al., 2017; Sarron et al., 2018; Tu et al., 2019). Some of the identified benefits of random forest is its capability to model complex variable interactions and prevent overfitting (Maxwell et al., 2018). Breiman (2001) found the random forest approach to perform better than other classifiers, including discriminant analysis, support vector machines, and neural networks. Similar to the statistical approach of bagging, the random forest approach is used to determine the optimal set of decision trees. Successive classification trees are independently constructed using a random sample of the data that does not depend on earlier decision trees (Johansen et al., 2015). The best split at each node is based on a subset of randomly selected predictor variables: in this case, set to 10 (mtry), or if the number of variables were fewer than 10, then all variables were considered. For those data sets with >10 variables, it was found through an iterative process that reducing the number of variables considered at each node to 10 did not affect the results. A value of 10 was selected because below that value, more variation in the accuracy between multiple runs of the models was noted. The final aggregation approach for all decision trees produced by the random forest algorithm prevents overfitting. A total of 1,000 decision trees (ntree) were used in this process to ensure stable predictions that were not too computationally intensive (Oliveira et al., 2012). Every training set is randomly sampled from the whole data set with replacement, i.e., the same observation can be used multiple times. Hence, in each decision tree, a bootstrap sample was selected, containing 63.2% of the data, with the remaining data used as evaluation data and to calculate the out-of-bag (OOB) error rate. In this study, the OOB error rate was used as an unbiased estimate of prediction error (Braga-Neto and Dougherty, 2004; Ghosh et al., 2014). Using a bootstrap sample for each decision tree further prevents overfitting. The experiments were implemented with the R package “randomForest” (https://CRAN.R-project.org/package=randomForest) (Liaw and Wiener, 2002).

Each of the six RGB and two multispectral image data sets were used independently to produce random forest models for predicting fresh shoot mass, fruit numbers, and yield mass per plant, using all plant observations, only salt-treated observations, and only control observations, yielding a total of 72 models (Johansen et al., 2019b). The models based on all plant observations were also applied to a subset consisting only of salt-treated plants and another subset including only control plants. This assessment was undertaken to evaluate how separate models adapted specifically to either salt-treated or control plants performed compared to those incorporating all plant observations. To determine the accuracy of each model, the importance of each predictor variable, the percentage of explained variance, and the root mean square error (RMSE) between the OOB observations of the field and UAV-derived measurements of fresh shoot mass, fruit numbers, and yield mass per plant were assessed. The relative root mean square error (rRMSE), defined as the RMSE divided by the mean values of the field observations, was also calculated. The decision trees were fully grown, and each was used to predict the OOB observations for that bootstrap sample. The final predicted value for an observation was the average of the OOB predictions for that observation based on the 1,000 decision trees. The permutation importance measure was used in this study. The importance of each variable is estimated by determining how much prediction error increases for each decision tree when OOB data for a selected variable is permuted and while all other variables are left unchanged. The increase in prediction error is then averaged over all trees and normalized by the standard deviation and measured as the percentage increase in mean squared error (%IncMSE) (Liaw and Wiener, 2002; Gregorutti et al., 2017).

## Results

### Object-Based Variable Importance for Predicting Biomass and Yield

**Figure 2:**
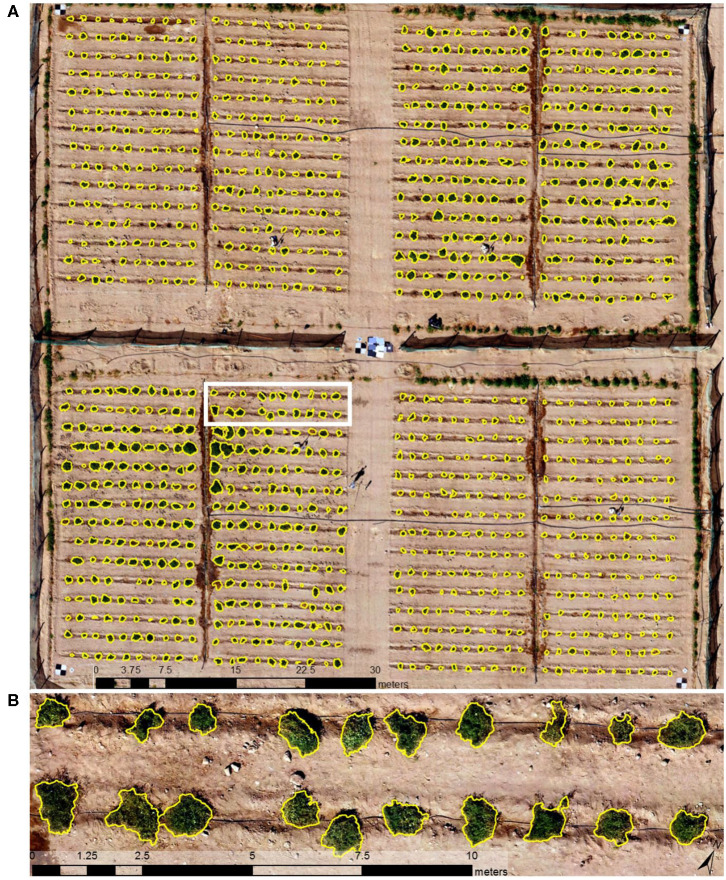
Delineation results (yellow outlines) based on unmanned aerial vehicle (UAV) imagery collected on January 7, 2018 for **(A)** the whole field site and **(B)** a subset of 20 tomato plants outlined in the white rectangle.

For the eight UAV image data sets (six RGB and two multispectral), 94.6–99.1% of all plants were automatically detected, with 5–16% of the plants subjected to additional manual adjustment to improve the delineation results. Plant length (longest axis) measured in the field with a tape measure produced an *R*^2^ value and RMSE of 0.85 and 0.052 m (*n* = 132), respectively, when compared with measurements from the automatically delineated plants. There was a tendency of smaller plants being slightly overestimated, whereas larger plants were slightly underestimated in length (Johansen et al., 2019a,b). Manual adjustment of the delineation results increased the *R*^2^ value to 0.97 and reduced the RMSE to 0.018 m. Figure 2 depicts the delineation results for January 7, and further details on the delineation results can be found in Johansen et al. (2019a).

Based on the delineated tomato plant objects, extracted parameters including shape features, spectral information, vegetation indices, texture features, and height information were used for predicting biomass and yield for each individual tomato plant. Table 4 shows the number of variables used for the random forest models, i.e., those variables with *R*^2^ > 0.10 when assessed against field-derived measurements of biomass and yield. When performing the random forest machine learning predictions of biomass and yield, the variable importance was calculated. As expected, some bands proved to be of high importance in most of the predictions, whereas other bands consistently appeared with low importance. When all models were rerun omitting the bands with low importance, it was found that reducing the number of bands with low importance did not increase the variance explained or reduce the RMSE. Hence, in this study, there was little benefit in further reducing the number of predictor variables included in the predictions.

**Table 4:** Number of variables used for predicting fresh shoot mass, fruit numbers, and yield mass from the six red–green–blue (RGB) and two multispectral (MS) unmanned aerial vehicle (UAV) data sets.

	**Jan 14 RGB/MS**	**Jan 7 RGB/MS**	**Dec 20 RGB**	**Dec 6 RGB**	**Nov 30 RGB**	**Nov 23 RGB**
Fresh shoot mass	20/31	20/35	19	14	14	8
Fruit numbers	20/31	18/26	15	14	8	4
Yield mass	20/31	19/27	15	14	8	8

**Figure 3:**
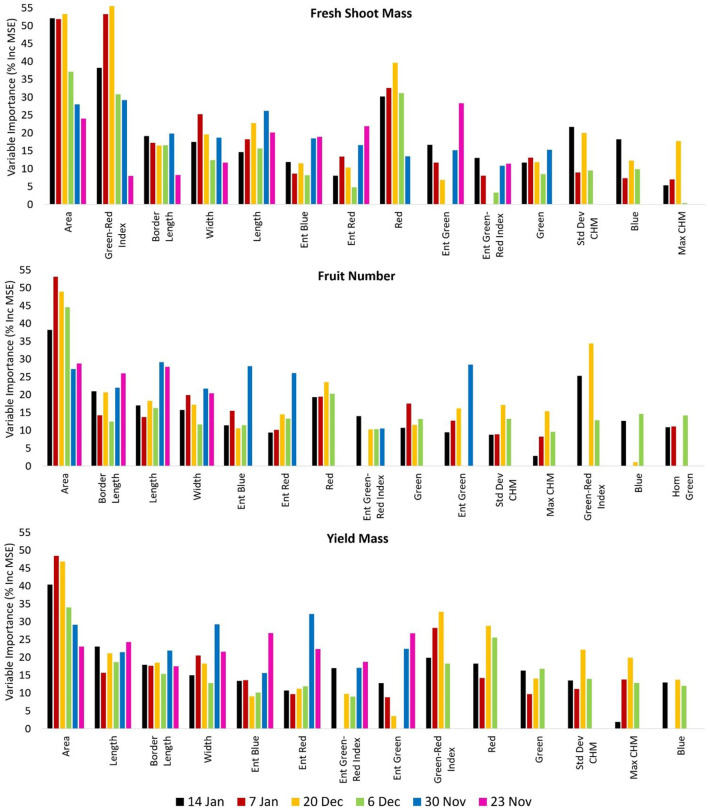
Variable importance in the random forest models based on the six unmanned aerial vehicle (UAV) red–green–blue (RGB) image data sets for the prediction of fresh shoot mass, fruit number, and yield mass. Only variables that were included in ≥3 out of the six RGB unmanned aerial vehicle (UAV) data sets are presented. Variables were sorted based on the number of data sets within which they were included and their importance value.

Prediction models of fresh shoot mass, fruit numbers, and yield mass were significantly improved by inclusion of shape features, including plant area, border length, width, and length, with plant area consistently achieving the highest importance values (Figure 3). However, there was a tendency of the importance scores for the plant area to reduce as a function of increasing time to harvest. Interestingly, the importance values for plant area were higher for the December 20 and January 7 data sets than the January 14 data set (closest to harvest), at least for fruit numbers and yield mass. After December 20, deterioration of many of the tomato plants occurred due to a number of destructive sandstorms on December 8 and 16, which damaged many plants (≈9%). That, combined with the increasing salt stress toward the end of the growing season, caused many of the remaining plants to exhibit signs of poor condition and senescence. Hence, the physical appearance and plant area of some plants may not have corresponded as well on January 14 to their measured yield as they did on December 20 and January 7. Plant area on December 20 and January 7 may reflect how well-plants coped with the sandstorms, with those coping well also yielding well. However, it is possible that the correlation between area and yield uncoupled in the final days of the experiment, i.e., January 14 (Figure 3), because of a lag between how well a plant performed at a particular time (e.g., measured by area) and how that translated to effects on fruiting and yield, which might not have manifested before January 14. The four shape features, i.e., plant area, border length, width, and length of individual plant objects, were important model input parameters for both the six RGB UAV collections (Figure 3) and the two multispectral UAV data sets (Table 5).

**Table 5:** Ranking of the 10 most important variables in the random forest models based on the two multispectral unmanned aerial vehicle (UAV) image data sets for the prediction of fresh shoot mass, fruit number, and yield mass.

**Importance**	**Fresh shoot mass**		**Fruit number**		**Yield mass**	
**Ranking**	**14-Jan**	**7-Jan**	**14-Jan**	**7-Jan**	**14-Jan**	**7-Jan**
1	Area	Area	Area	Area	Area	Area
2	Width	Width	Length	Width	Length	Width
3	Length	NIRRENDVI	Ent RedEdge	Ent RedEdge	Ent RedEdge	Ent RedEdge
4	NDVI	Length	Width	RENDVI	Width	NIRRENDVI
5	NIRRENDVI	RENDVI	Green-Red index	Green-Red index	Green NDVI	Green-Red index
6	Green-Red index	Green-Red index	Ent NIR	NIRRENDVI	Dis NDVI	RENDVI
7	Red	Red	Green NDVI	Length	Border length	Length
8	RENDVI	Hom NDVI	Ent green	Hom NDVI	Con red	Hom NDVI
9	Border length	Ent Green	Border length	Border length	Ent NIR	Green NDVI
10	Green NDVI	Ent RedEdge	Dis NDVI	Dis NDVI	Ent green	Border length

In terms of spectral metrics, the Green–Red Index had high importance for predicting fresh shoot mass for all six RGB UAV captures (Figure 3) as well as the multispectral UAV imagery (Table 5). Several of the other multispectral vegetation indices were also highly ranked in their importance for predicting fresh shoot mass, fruit numbers, and yield mass. In fact, for the prediction of fresh shoot mass on January 14 using the multispectral UAV imagery, the 10 highest ranked predictor variables in terms of importance consisted entirely of shape features and vegetation indices (Table 5). Many UAV-based crop studies have reported positive correlation between vegetation indices and both biomass and yield (e.g., Hassan et al., 2019; Niu et al., 2019; Zheng et al., 2019). In our study, the Green–Red Index showed relatively lower importance for the prediction of fruit numbers and yield mass based on the RGB UAV imagery. Again, this may have been attributed to the severe sandstorms prior to the December 20 data collection, which could have limited the Green–Red Index from providing greater predictive value in terms of fruit numbers and yield mass. In addition, some senescent tomato plants still produced high fruit numbers at the end of the growing season, which again also would have reduced the capacity of vegetation indices successfully predicting fruit numbers and yield mass. It should be noted that the Green–Red index achieved the highest importance in predicting fresh shoot mass on December 20. This date corresponds to the highest recorded green biomass during the season, as the damage from the preceding sandstorm events and increasing salt-stress resulted in the senescence of many plants after December 20 (Johansen et al., 2019a).

Several of the texture features extracted for the individual tomato plants were of importance for predicting fresh shoot mass, fruit number, and yield mass from the RGB and multispectral UAV imagery. The entropy texture measure of the spectral bands and vegetation indices proved especially useful for these predictions. The entropy texture measure tended to show an exponential relationship with biomass and yield. Entropy texture extracted from a gray level co-occurrence matrix measures the spatial disorder of pixels, with texturally uniform image objects having small values (Kekre et al., 2010). Tomato plants with increasing biomass and yield appeared with increasing entropy texture values, indicating a more complex and “disorderly” plant canopy architecture. However, once the biomass and yield reached a set threshold in size, the entropy texture values became unsuited for accurately predicting biomass and yield because of the exponential relationship. Hence, large plants with large amounts of biomass and high fruit numbers and yield mass represented the maximum spatial disorder of pixels accounted for by the entropy measure. While the entropy texture measure might be a useful predictor variable for smaller tomato plants with limited biomass and yield, the measure became increasingly unsuited as a predictive variable with increasing biomass and yield. This is likely the reason why the entropy texture measure was found informative during the earliest image campaigns (i.e., when the plants were still small) but had a lower importance for those data sets collected closer to harvest (i.e., when the plants were larger) (see Figure 3).

The measurements of plant height and their standard deviation was in most cases identified with low importance as a predictor variable. A cause of this might have been the fact that plant height did not vary much between large and small plants, with plant growth mainly occurring horizontally. In addition, the sandstorm events that occurred during the growing season caused many of the branches to break, which also affected plant height in some cases. The importance of predictor variables was also assessed separately for the control and salt-treated tomato plants. While variations in the ranking of predictor variables occurred, the shape features were still found to be most important, followed by vegetation indices and then texture measures (the entropy texture measure in particular). However, one noticeable variation was the much higher importance of the standard deviation of maximum plant height for the salt-treated tomato plants when using the RGB UAV imagery. This may have been due to the much lower yield and hence limited effect of the tomato fruit weight on the plant height. It is likely that the standard deviation of plant height played an important role in the salt-treated plants because of their smaller plant size and lower degree of sprawling. Hence, plant height was a stronger determinant of plant size overall, compared with the control plants.

### Prediction of Biomass and Yield of All Plants

#### Distribution of Predictions for All Plants

**Figure 4:**
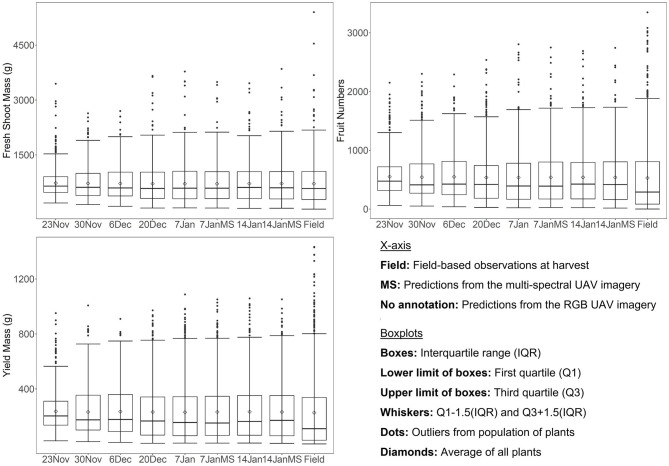
Box-and-whisker plots, showing the variation in red–green–blue (RGB) and multispectral unmanned aerial vehicle (UAV) predicted fresh shoot mass, fruit numbers, and yield mass throughout the growing season in relation to field-based observations at the time of harvest.

The average field-derived fresh shoot mass, fruit numbers, and yield mass for all plants remaining at harvest was 715.29, 532.06, and 226.96 g, respectively, whereas those for the control plants were 1070.67, 810.00, and 362.55 g, respectively, while those of the salt-treated plants were 355.56, 241.89, and 87.47 g, respectively. When assessing the field-based average values against those predicted from all eight UAV image data sets (November 23-January 14) using the random forest models developed on (and applied to) all plants, it was found that all the average predicted values were within 16.97 g (2.37%), 20.12 (3.78%), and 9.59 g (4.23%) for fresh shoot mass, fruit numbers, and yield mass, respectively (Figure 4). This indicates that the average biomass and yield can be predicted with high accuracy as much as 8 weeks in advance, even with the disruptive sandstorms and increasing salt-stress affecting those plants remaining at harvest. However, the range of values for the field observations was much larger than from the predictions of the earliest UAV data collections (e.g., see interquartile range between field observations and predictions for November 23 in Figure 4). The whisker and interquartile range of fresh shoot mass, fruit numbers, and yield mass predictions approached those of the field observations the closer to harvest the UAV data were collected (Figure 4). Therefore, while the average fresh shoot mass, fruit numbers, and yield mass could be accurately mapped well in advance of harvest, the prediction accuracy for an individual plants' fresh shoot mass, fruit numbers, and yield mass increased as a function of decreasing time until harvest. For all eight UAV data sets, the median of the predicted fruit numbers and yield mass was overestimated (Figure 4): although this appeared to be less of an issue for the predicted fresh shoot mass. It can also be seen from Figure 4 that those plants with very high fresh shoot mass, fruit numbers, and yield mass were underestimated based on the UAV imagery.

#### Distribution of Predictions for Control and Salt-Treated Plants

When assessing the average values of predicted fresh shoot mass, fruit numbers, and yield mass for just the control plants using the random forest models developed on all plants, the first three UAV data sets (November 23 and 30, December 6) significantly underestimated average values of 108.76–238.14 g (10.16–22.24%) for fresh shoot mass, 52.14–175.87 (6.44–21.71%) for fruit numbers, and 30.00–87.85 g (8.27–24.23%) for yield mass (Table 6). Assessing just the salt-treated plants showed the opposite pattern, with the first three UAV data sets (November 23 and 30, December 6) producing significantly overestimated average values of 121.14–272.58 g (34.07–76.66%) for fresh shoot mass, 92.27–221.76 (38.15–91.68%) for fruit numbers, and 47.67–110.05 g (54.50–125.81%) for yield mass. Hence, the underestimation of average values for the control plants and the overestimation of the salt-treated plants balanced the average values for predicting fresh shoot mass, fruit numbers, and yield mass for all tomato plants for the first three UAV data captures. It also shows that while the overall average biomass and yield for all plants were accurately predicted, the predictions were not accurate for individual plants. This was likely because the response to the salt treatment had not yet come into full effect on November 23 and 30 and December 6, and hence, the predictions did not account for the further increase in salt treatment and consequent impact on plant growth. On the other hand, biomass and yield of control plants were underestimated as the initial impact of the salt treatment on half the plants reduced the overall average. The data sets collected on December 20 and January 7 and 14 produced much better average estimates for the control-only and salt-treated-only data sets, with all being within 14% of the average value estimated from the field data. This was because the variables extracted from the UAV imagery on those dates and used for predicting biomass and yield at harvest better represented each individual plant's condition at harvest.

**Table 6:** Percentage average over- and underestimation of predicted fresh shoot mass, fruit numbers, and yield mass in relation to the field measurements when assessing control and salt-treated plants separately.

	**14-Jan MS**	**14-Jan**	**7-Jan MS**	**7-Jan**	**20-Dec**	**6-Dec**	**30-Nov**	**23-Nov**
Fresh shoot mass—control (%)	−1.53	−2.04	−2.76	−2.45	−4.36	−10.16	−12.72	−22.24
Fresh shoot mass—salt (%)	7.03	6.74	7.19	6.04	12.49	34.07	42.84	76.66
Fruit numbers—control (%)	2.84	3.12	−0.44	−0.61	0.24	−6.44	−12.29	−21.71
Fruit numbers—salt (%)	−1.50	−1.00	10.22	8.62	6.15	38.15	52.58	91.68
Yield mass—control (%)	2.01	2.34	2.21	1.96	−0.66	−8.27	−14.24	−24.23
Yield mass—salt (%)	3.54	4.82	1.37	−0.22	13.79	54.50	73.81	125.81

*Positive values indicate overestimation and negative values indicate underestimation*.

#### Assessment of All Individual Plants From Single Date UAV Data

The initial assessment of prediction accuracies of fresh shoot mass, fruit numbers, and yield mass was based on the OOB observations of tomato plants, including both the control and the salt-treated plants. The RGB image-based results showed that in the week before harvest, the explained variance of fresh shoot mass, number of tomatoes, and yield mass were 86.60% (RMSE = 208.4 g, rRMSE = 29.14%), 59.46% (RMSE = 379.7, rRMSE = 71.36%), and 61.09% (RMSE = 168.9 g, rRMSE = 74.40%), respectively. Two weeks before harvest, the explained variance was slightly higher and the RMSE slightly lower than the week prior to harvest for the predicted fresh shoot mass, fruit numbers, and yield mass, using the RGB imagery (Table 7), possibly attributed to deterioration of some plants toward the end of the growing season. The results derived from the multispectral and RGB UAV data sets collected on January 7 and 14 were very similar and will be further compared in *Comparison of Model Results for Control and Salt-Treated Plants* below. On December 20, the explained variance of fresh shoot mass, fruit numbers, and yield mass was 79.20% (RMSE = 259.8 g, rRMSE = 36.31%), 55.90% (RMSE = 395.5, rRMSE = 74.33%), and 57.73% (RMSE = 175.7 g, rRMSE = 77.42%). On December 6, a reduction of >20% in explained variance and associated increases in RMSE was observed when comparing with December 20 for all three variables. As destructive sandstorms damaged many plants (≈9%) on December 8 and 16, the imagery collected after the sandstorm events provided more representative information on biomass and yield at harvest for the individual plants than the data collected prior to the sandstorms (Johansen et al., 2019b). The data collected on November 23 and 30 were found to be unsuitable for predicting biomass and yield at harvest for individual plants. Although the explained variance of fresh shoot mass was 46.57% on November 30, the RMSE was still more than double those for January 7 and 14.

**Table 7:** Percentage explained variance (EV) and relative root mean square error (rRMSE) based on predictions of fresh shoot mass, fruit numbers, and yield mass for all eight unmanned aerial vehicle (UAV) data sets based on the joint analysis of control and salt-treated tomato plants.

	**14-Jan MS**	**14-Jan**	**7-Jan MS**	**7-Jan**	**20-Dec**	**6-Dec**	**30-Nov**	**23-Nov**
EV fresh shoot mass (%)	87.69	86.6	87.95	87.61	79.2	59.14	46.57	17.77
rRMSE fresh shoot mass (%)	27.90	29.14	27.64	28.02	36.31	50.89	58.23	72.21
EV fruit numbers (%)	57.86	59.46	63.88	60.61	55.9	33.77	25.3	2.15
rRMSE fruit numbers (%)	72.89	71.36	67.37	70.25	74.33	91.20	96.77	110.79
EV yield mass (%)	59.47	61.09	66.51	64.37	57.73	37.51	26.36	6.31
rRMSE yield mass (%)	76.08	74.40	69.09	71.09	77.42	94.27	102.25	115.36

*The two multispectral UAV data sets are denoted with “MS,” whereas the other data sets represent the RGB UAV data*.

**Figure 5:**
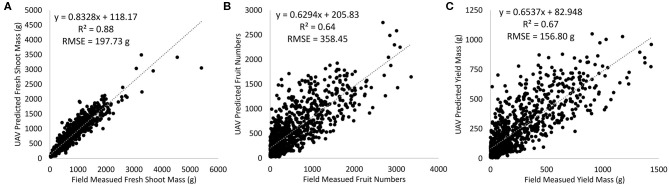
Scatterplots showing the linear relationships between field-measured and predicted **(A)** fresh shoot mass, **(B)** fruit numbers, and **(C)** yield mass based on the multispectral unmanned aerial vehicle (UAV) imagery collected on January 7, 2018.

Figure 5 provides an example, based on the multispectral UAV imagery from January 7, of the distribution of predicted fresh shoot mass, fruit numbers, and yield mass in relation to the field-based measurements. The best-fit regression lines show that there was a tendency of small plants being overestimated in terms of predicted fresh shoot mass, fruit numbers, and yield mass. However, those plants with high fresh shoot mass, fruit numbers, and yield mass had their values underestimated. The higher explained variance for fresh shoot mass was related to the ability to integrate shape features such as plant area and other plant dimensions. Those shape features and the greenness of the plants, expressed through the use of vegetation indices, are closely related to biomass (Bendig et al., 2015). Fruit numbers and yield mass have a more indirect relationship to the shape and greenness of the plants. For example, some smaller and senescent field-assessed tomato plants were identified with large numbers of fruit, which would likely have been influenced by the different types of accessions and the two treatments.

**Figure 6:**
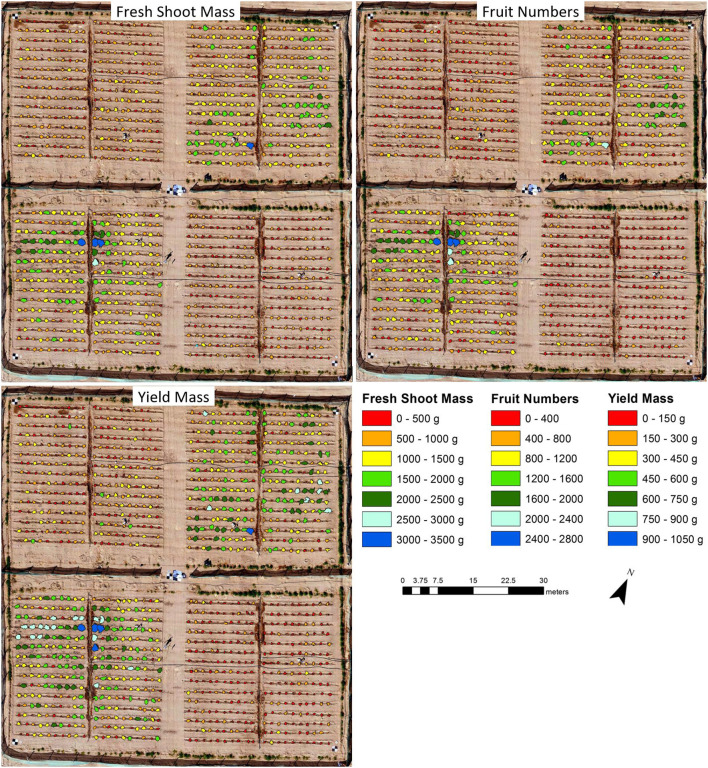
Maps of predicted fresh shoot mass, fruit numbers, and yield mass per tomato plant based on the multispectral unmanned aerial vehicle (UAV) imagery collected on January 7.

Figure 6 clearly shows the difference between the control and salt-treated tomato plants in terms of fresh shoot mass, fruit number, and yield mass based on the multispectral UAV imagery collected on January 7. Most of the plants (96.7%) in the salt-treated plots had a fresh shoot mass between 0 and 1,000 g, with only 3.3% plants appearing in the category between 1,000 and 1,500 g. Although ~48% of the control plants appeared in the category between 0 and 1,000 g of fresh shoot mass, ~48 and 4% occurred with fresh shoot mass weights between 1,000 and 2,000 g and 2,000 and 3,500 g, respectively. The predicted distribution in terms of fruit number and yield mass between control and salt-treated plants was similar to that of fresh shoot mass, with all but six and five plants appearing with >800 fruits and >450 g of yield mass, respectively, for the salt-treated plots. The control plots on the other hand produced fruit numbers and yield mass for some plants up to 2,751 fruits and 1,050 g of fruit, respectively.

#### Assessment of All Individual Plants From Multitemporal UAV Data

Combining data from different dates was also investigated to assess if the use of multitemporal information improved the prediction accuracies of fresh shoot mass, fruit numbers, and yield mass. Using the predictor variables from the multispectral imagery from both January 7 and 14, the explained variance increased by 1.66% (from 87.95 to 89.61%) and 0.10% (from 63.88 to 63.98%), and the rRMSE decreased by 1.94 and 0.04% for fresh shoot mass and fruit numbers, respectively, in comparison to the results using only data from January 7. For the yield mass predictions, the use of multispectral data from both January 7 and 14 caused a decrease in the explained variance of 0.64% and an increase in rRMSE of 0.76%. For the RGB imagery, the combination of data from January 7 and 14 and December 20 produced increases in explained variance of 1.44 (from 87.61 to 89.05%), 2.31 (from 60.61 to 62.92%), and 1.45% (from 64.37 to 65.82%) and decreases in rRMSE of 1.67, 2.01, and 1.36% for fresh shoot mass, fruit numbers, and yield mass, respectively. The addition of RGB data from November 23 and 30 reduced the prediction accuracies. As such, negligible differences in prediction accuracies were identified from using multitemporal data as opposed to a single date (January 7 or 14) prior to harvest.

### Comparison of Model Results for Control and Salt-Treated Plants

**Figure 7:**
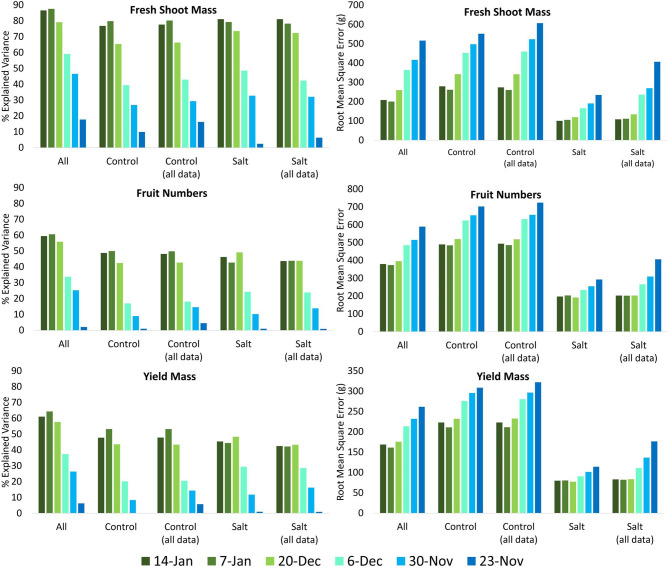
Percentage explained variance and root mean square error of predicted measurements of fresh shoot mass, fruit numbers, and yield mass based on the RGB unmanned aerial vehicle (UAV) imagery collected on November 23 and 30, December 6 and 20, and January 7 and 14 using random forest models based on (1) all tomato plants, (2) only control plants, (3) all plants but applied only to the control plants, (4) only salt-treated plants, and (5) all plants but applied only to the salt-treated plants.

Different random forest models were developed for (1) all tomato plants, (2) the control plants only, and (3) the salt-treated plants only. The models developed for all tomato plants were applied to all plants, the control plants only, and the salt-treated plants only, whereas the models developed for the control plants only and the salt-treated plants only were only applied to those specific subexperiments. As a general trend, the variance explained decreased and the rRMSE increased as a function of increasing time until harvest, indicating that approaching harvest time, the predictions of fresh shoot mass, fruit numbers, and yield mass improved (Figure 7). The random forest model developed on and applied to all (i.e., both control and salt-treated) tomato plants produced the highest proportion of explained variance and the lowest rRMSE for predicting fresh shoot mass, fruit numbers, and yield mass on December 20, January 7 and 14 (Figure 7). It is likely that the larger range of measurements included when using both control and salt-treated plants in the model development improved the prediction, as the salt-treated and control plants included some of the plants with the lowest and highest measures, respectively, of fresh shoot mass, fruit numbers, and yield mass.

#### Assessing Models Developed for Control Plants Only and Salt-Treated Plants Only

The random forest models developed on and applied to just the control plants had very similar amounts of explained variance (within 0.011) to the results produced when using the random forest model developed for all plants and applied only to the control plants for the UAV data sets collected on December 20 and January 7 and 14. On December 6, a reduction in explained variance of 3.49% was observed when using the model developed on and applied to just the control plants. However, for fresh shoot mass, fruit numbers, and yield mass, the explained variance was higher (by up to 6.29 on December 6, 5.28 on December 20, and 5.05% on December 20, respectively) between December 6 and January 14, when using the random forest models specifically developed on and applied to only the salt-treated plants compared to using the model developed on all plants (Figure 7). In addition, within the same period, a lowering of the RMSE was observed for predicted fresh shoot mass, fruit numbers, and yield mass of 6.06–70.15 g (1.70–19.73%), 5.27–31.05 (2.18–12.84%, excluding January 7), and 1.95–20.33 g (2.23–23.24%), respectively, using the model for the salt-treated plants only (Figure 7). These results indicate that models to predict biomass and yield of salt-stressed plants may need to be separately developed, whereas predicting the yield of control plants was not affected by the inclusion of salt-stressed plants within the models. The results also demonstrate the ability to predict biomass and yield of individual tomato plants up to 4 weeks before harvest.

#### Comparing Model Results of the Multispectral and RGB UAV Imagery

**Figure 8:**
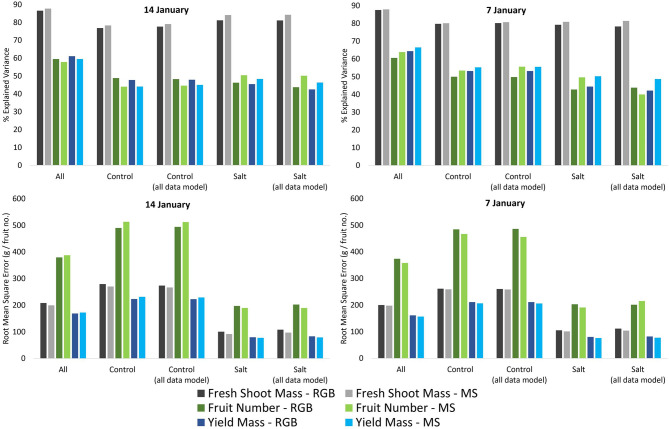
Comparison between the percentage explained variance and root mean square error of predicted measurements of fresh shoot mass, fruit numbers, and yield derived from the multispectral and red–green–blue (RGB) unmanned aerial vehicle (UAV) imagery acquired on January 7 and 14, using random forest models based on (1) all tomato plants, (2) only control plants, (3) all plants but applied only to the control plants, (4) only salt-treated plants, and (5) all plants but applied only to the salt-treated plants.

The difference in explained variance between the multispectral and RGB UAV-derived predictions of fresh shoot mass, fruit numbers, and yield mass was within 3.27% for both January 7 and 14 when assessing all plants. While the multispectral UAV data produced predictions with slightly higher explained variance for all variables than the RGB imagery collected on January 7, the RGB UAV imagery produced slightly higher explained variance for fruit numbers (1.60%) and yield mass (1.62%) for all plants on January 14 (Figure 8). Corresponding lowering of the RMSE on January 7 for all three variables predicted from the multispectral UAV imagery was observed, while the multispectral UAV-based RMSEs for fruit numbers and yield mass on January 14 increased slightly (8.14 and 3.81 g, respectively). An objective comparison of the results from the multispectral and RGB imagery was difficult to achieve because of the differences in suitable predictor variables (Table 2), number and wavelength locations of spectral bands, pixel sizes (0.50 cm for RGB and 1.12 cm for multispectral), camera specifications (12 MP for RGB vs. 1.2 MP for the multispectral), as well as field of view and focal length differences, among others. Despite this, Figure 8 shows that neither multispectral nor RGB UAV imagery were clearly advantageous in any combination of variables, models, and treatments, as long as accurate delineation of tomato plants can be achieved for derivation of plant shape features, as these were the most important variables for the prediction of biomass and yield.

For instance, when comparing the multispectral and RGB UAV imagery for the control plants only, the RGB imagery explained the highest amount of variance for fruit numbers and yield mass using both the random forest model based on the control plants (3.72–4.81% higher) and on all plants (2.89–3.65% higher) on January 14 (Figure 8). In contrast, the multispectral imagery could be used to predict fresh shoot mass with an explained variance of 1.43 and 1.46% higher than the RGB imagery for the control plants using control plants only and all plants for modeling, respectively. On January 7, the multispectral UAV imagery produced the highest proportion of explained variance in all cases for the control plants (0.32–5.83% higher than the RGB imagery), with the highest difference of 5.83% for fruit numbers when using a model developed on all plants and applied only to the control plants (Figure 8).

When predicting the salt-treated plants only based on the random forest model specifically developed on only the salt-treated plants, the multispectral UAV imagery produced higher proportions of explained variance than the RGB UAV imagery for both January 7 (higher by 1.66% for fresh shoot mass, 5.82% for yield mass, and 6.89% for fruit numbers) and 14 (higher by 2.90% for yield mass, 3.01% for fresh shoot mass, and 4.13% for fruit numbers). Similar observations were identified when using the random forest model based on all plants for salt-treated plants to predict their fresh shoot mass, fruit numbers, and yield mass, where the multispectral UAV imagery explained the highest amount of variance (3.13–6.51% higher) for both January 7 and 14, with the exception of fruit numbers on January 7, when the RGB UAV imagery (43.86%) explained 3.88% more variance than the multispectral UAV imagery (39.98%) (Figure 8). Overall, the increase in explained variance were generally accompanied by a decrease in RMSE.

## Discussion

### Model Transferability of UAV Data

Using the random forest machine learning approach, our results showed that UAV imagery collected within 4 weeks of harvest provided the best results for predicting biomass and yield at harvest for individual tomato plants. Our results also indicated that separate random forest models for predicting yield of salt-stressed plants might be required. In contrast, the yield of control plants could be predicted with similar accuracies when using models developed on both all plants and control plants only. This may be attributed to the fact that the control plants covered a similar range of values to that for all plants, whereas the salt-treated plants mainly covered the lower range of recorded values. For instance, 157 (out of 497) control plants had higher numbers of fruit yield than any of the salt-treated plants. Hence, those models developed specifically on and applied to the salt-treated plants performed better than using those developed on all plants when applied to the salt-treated plants. This emphasizes the importance of carefully selecting data sets in terms of size, variability, and representativeness for model development to ensure transferability (Liu et al., 2018; Maxwell et al., 2018; Ma et al., 2019). Model transferability will also require data that are representative in other contexts, e.g., including multiple growing seasons, different areas, different climate and weather conditions, different soils, and different management practices (Maxwell et al., 2018). Hence, future work should focus on assessing the model transferability of machine learning approaches for UAV-based mapping applications. It will be valuable for growers to know if the same machine learning model can be used under different contexts or if the same model can be used universally for the same plant species.

The analysis included 199 *S. pimpinellifolium* accessions and one *S. lycopersicum* accession (the commercial tomato, Heinz 1706). Considering the high mapping accuracies of the UAV data collected in the weeks preceding harvest, the developed models did not seem to be affected by the large variety of accessions included in the training data. The three salt-treated *S. lycopersicum* plants all died prior to harvest, and while the biomass of the three control plants was predicted (based on the multispectral UAV imagery collected on January 7) to be within 6.84–14.48% of the field-based observations (better than the RMSE and rRMSE of 197.7 g and 27.64%, respectively, Figure 5A), the predicted yield was significantly overestimated, with two plants not producing fruit and one plant producing 630 fewer fruits and 283 g of yield mass less than predicted. Despite the small sample size of the *S. lycopersicum* species, these results raise questions on whether the developed models can be employed for different tomato plant species. Hence, future studies testing model transferability of machine learning approaches should also test different species.

### The Issue of Data Dimensionality and Errors

The availability of UAV data for supporting smart farming is expected to grow significantly in the future (Wolfert et al., 2017; Liakos et al., 2018). The dimensionality of available data is also expected to increase, with the availability of hyperspectral imagery providing hundreds of spectral bands for analysis (Torresan et al., 2016; Adao et al., 2017). Likewise, additional sensors provide new observation capacity, e.g., LiDAR data providing three-dimensional information on crops, or thermal data providing temperature measurements at high spatial resolution, both of which are increasingly being integrated with other information and used for crop assessment (Calderon et al., 2013; Ivushkin et al., 2019). The growing availability of such UAV-based data sets will likely make predictions of biomass, yield, and other biophysical and biochemical properties not only more accurate but also more complex. In many instances, this results in higher computational costs and longer processing times, which limits the proficiency of real-time delivery. With the increasing UAV data dimensionality, machine learning approaches become the only feasible option for big data analytics. In most cases, preprocessing approaches (standard UAV processing workflow to produce orthomosaics, geo-referencing, radiometric corrections, etc.) are time consuming for large data sets. To increase the ability to achieve well-calibrated and analyzed near real-time UAV map outputs from big data, machine learning approaches suitable for converting raw data into final map outputs should be explored (Yang et al., 2020).

While deep learning models are designed for high-quality data feature learning (Zhang et al., 2018), some research has experimented with deep learning models for low-quality data. Approaches such as those by Vincent et al. (2010) and Wang and Tao (2016) have focused on data denoising routines and identifying reliable features within corrupted data, and these might be explored in future research for deep learning models applied to multiple UAV data sets collected under various conditions and with different acquisition parameters. Zhang et al. (2018) also discussed a multimodel deep learning approach specifically suited to heterogeneous data, which might be explored in future research as well to regionalize UAV data sets for optimization of analysis and results. Despite the availability of some approaches suited for reducing the effects of uncertainties and noise inherent in UAV data, further exploration is still required to effectively reduce processing time and alleviate the need for complex intermediate processing steps of UAV data, preventing near real-time delivery of information on crop variables. For instance, uncertainty in UAV-based thermal data may be introduced by wind speed, wind direction, and flight direction. UAV optical data collected by RGB and multispectral and hyperspectral sensors are all sensitive to the time, season, and latitude of data acquisition, as that will affect the solar angle and shadowing effects. In addition, the quality of sensor calibration may impact data quality (Barreto et al., 2019). The current basic preprocessing chain of optical UAV data includes many steps, where multiple filtering modes, blending modes, color correction, and interpolation approaches will affect the orthomosaic and hence potentially introduce data noise. In addition, the spectral and radiometric properties of different camera systems tend to differ, making direct comparison of data unfeasible (Tmusic et al., 2020). In addition to all of these potential uncertainties, flight planning parameters, such as forward overlap, sidelap, speed, flight direction, and flying height (Tu et al., 2020), as well as weather conditions during data acquisition, e.g., wind speed and direction, cloud shadows, dust near the ground, and variations in the atmosphere's composition (Zhang et al., 2014; Ziliana et al., 2018), will all influence and affect the quality of the acquired data. With all of these potential issues introduced inherent in UAV data, it is important that future work explore the sensitivity of machine learning approaches to these uncertainties.

### Variable Standardization of Model Inputs

Ultimately, our research showed that plant-based shape parameters had the highest importance for predicting biomass and yield of the tomato plants using random forest models. As long as the UAV-derived imagery is geometrically registered and a suitable delineation approach can be developed, the mapping of shape parameters is likely to be influenced less than the spectral characteristics when using cameras with different spectral and radiometric characteristics or if flight planning parameters vary between data acquisitions. Hence, if integrating multiple data sets from different UAV-based sources for mapping tomato plants, shape parameters should be a main focus to ensure consistency between data sets. Vegetation indices proved useful in this research as well, and they have been utilized previously to reduce atmospheric effects and limit the need for image calibration (Lillesand et al., 2015; Xue and Su, 2017; Fernandez-Gallego et al., 2019). However, ratio-based indices are still affected by the spectral resolution and band width of the sensor used. Vegetation indices often fail to account for canopy-background interactions and canopy bidirectional reflectance anisotropies, especially those associated with shadowing effects, and become insensitive to vegetation with high leaf area index values, which leads to insensitivities to vegetation variation of dense plants and trees (Gitelson et al., 1996; Sims and Gamon, 2002a; Asner and Warner, 2003). As such, the application of vegetation indices for mapping biophysical and biochemical parameters lacks generality, making them time, space, and crop type specific (Houborg et al., 2007). Mapping actual biophysical and biochemical parameters from optical data and using these as model inputs for predicting crop parameters such as biomass and yield might ensure further standardization when integrating data collected for different areas and from different sensors with machine learning approaches (Houborg et al., 2007; Gholizadeh et al., 2015). That will generally require field-based measurements to be collected for model calibration purposes. In fact, the use of high-quality field data is imperative for both calibration and validation to standardize UAV-derived outputs suited as model input for prediction of crop parameters (Von Bueren et al., 2014). Radiometric correction of imagery is equally important to ensure consistency of optical image data over time and between sites (Jeong et al., 2018; Tmusic et al., 2020). Hence, despite the large array of UAV image data sets and acquisition and processing routines for crop assessment, potential avenues exist to improve the consistency of diverse data used as input for machine learning approaches.

## Conclusions

A novel approach for using UAV-based imagery collected at various intervals prior to harvest was employed to predict fresh shoot mass, fruit numbers, and yield mass of tomato plants at harvest using a random forest machine learning approach. Shape features derived from individual tomato plants were determined to be the most important predictor variables, followed by vegetation indices and image texture. While the average biomass and yield at the field level could accurately be predicted up to 8 weeks prior to harvest, a significant reduction in prediction accuracy of biomass and yield of individual plants was identified when using UAV imagery collected more than 4 weeks before harvest. This was attributed to sandstorm events, where the imagery collected after the sandstorms provided more representative predictions of biomass, fruit numbers, and yield mass at harvest for individual plants than the data collected prior to the sandstorms. Models specifically developed for predicting yield from the salt-stressed plants increased the explained variance by up to 6.29% (relative to those models for all plants), whereas little (<1.1% explained variance) variation occurred in the results for predicting yield of the control plants irrespective of which models were used.

The research demonstrates the suitability of using UAV imagery and a random forest machine learning approach for biomass and yield prediction of tomato plants but highlights the need for careful consideration in terms of data inputs (e.g., parameters of control vs. salt-stressed plants) for model development. It is important to be mindful of data quality both with regard to UAV-based imagery and field-based observations, as machine learning approaches are inevitably influenced by errors. It is therefore imperative to follow standardized procedures when extracting data used as input into machine learning algorithms. Future work should compare the results of different machine and deep learning approaches for predicting biomass, yield, and other biophysical and biochemical properties of agricultural crops and explore the sensitivities of these approaches to typical UAV data inconsistencies (preprocessing steps, cloud shadow contamination of imagery, sensor noise, etc.). Assessing the transferability of developed machine and deep learning models to test their applicability to a wider context also needs to be explored (e.g., for UAV imagery collected of the same crops but for different areas or during different growing seasons for the same area). Finally, heritability calculations to evaluate the variation between phenotypic traits of the tomato plants in response to genetic variation among the different tomato plant accessions will be an important extension of this research. This research and identified future directions may provide growers with valuable UAV-derived information on how to manage plant growth, increase yield, and obtain advance knowledge on harvesting, sales, and distribution requirements of tomatoes and other fruit.

## Data Availability Statement

The datasets generated for this study are available on request to the corresponding author.

## Author Contributions

MT conceived the whole plant experiment and was involved in all aspects of the project. MFM designed the UAV-focused aspects of the experiment, as well as associated ground-based data collections, and was involved in all aspects of the project. KJ undertook all UAV image processing and analysis and led the writing of the manuscript, with MFM, MT, and MJLM also contributing. MJLM and MT designed the ground-based component of the plant experiment and, with GF, SN, and MAAM, coordinated the experiment, including field data collection and the final harvest. KJ, YM, BA, SA-M, MZ, and YA were responsible for field equipment and collection of the UAV imagery and field data. MAAM led a team of workers to undertake planting, irrigation, fertilization, observation, and washing of plants after sandstorms, and harvesting.

## Conflict of Interest

The authors declare that the research was conducted in the absence of any commercial or financial relationships that could be construed as a potential conflict of interest.
